# A Model Hospital

**Published:** 1891-12-05

**Authors:** Conrad W. Thies

**Affiliations:** Secretary to the Royal Free Hospital


### A Model Hospital.

By Conrad W. Thies, Secretary to the Royal Free Hospital.

*(Concluded from page 108.)*

I may mention here that four small pavilions are specially set apart for the isolation of all new cases, which are kept under observation for twenty-four hours before being admitted into the general wards. Every possible precaution is taken to prevent germs being carried. To this end the medical officers, male attendants, and convalescent patients wear long linen blouses over their garments. I could not fail to be struck with the constant evidence I saw of the endeavour which is made to carry out the principles of asepsis even in the most minute details, and I was assured that the medical and surgical results of the adoption of all these elaborate precautions fully justified their use.

The foregoing description applies to all the large single-storeyed pavilions, as they are constructed upon an almost identical plan. There are, however, some smaller pavilions adapted for special purposes, which present certain deviations which I need not describe

I was greatly impressed by the bath pavilion, with its complete equipment of baths of every description. In the upper wards I saw patients who had been immersed for many weeks in bath beds of special construction. The delirium tremens pavilion has a ward on the ground floor for six patients who are kept under constant observation; also six solitary cells for violent cases; special rooms are provided for paying patients and for those who are suspected of insanity. Four double-storeyed pavilions are set apart for patients who can afford to pay for their treatment.

The operating pavilion occupies a central position in the midst of the surgical wards; it is a two-storeyed building, and has cellars beneath. On one side of the main corridor is the large operating theatre; it is lighted by an octagonal bow window, constructed of an iron framework with double windows to exclude any draughts of air. The floor is of terazzo, and made to incline gently, so that all discharges can readily be washed into the drains. The walls are covered with glazed tiles. The floor is warmed by steam pipes, and in addition there is a system of wall heating. I was informed that when the outside temperature has been down to zero it has been easy to maintain the theatre at a uniform temperature of 80 deg. Fahr.

The mortuary consists of a large building which is divided into two parts each surrounded by its own separate court-yard. One portion is occupied by the chapel, with rooms for mourners and friends. There is a special entrance to the chapel from a side street. The other part is devoted to the pathological department, and consists of museum, laboratories, microscopical rooms, &c., also a large post-mortem room, which is lighted by eight windows and has, in addition, one side, 26 feet wide, entirely composed of glass in an iron framework. It is fitted with eight tables, and there is a special lift for bringing the bodies up from the refrigerating chamber beneath. The floor is of terazzo, the drains are carried into a special cesspool, where all discharges are disinfected before being allowed to enter the ordinary sewers. The administrative building, the domestic and nurses' department do not call for any special description as they are of the usual type, but I may say that they have been arranged and fitted up with the careful attention to details which characterises the whole of the institution.

The following particulars respecting the entire cost of the hospital is of interest, especially when a comparison is made with the cost of some of our recently-built London hospitals:—

| | | |
|---|---|---|
| The land was valued at £13,000= | £9 14 0 | per bed. |
| The buildings, &c., cost 253,500= | 189 3 6 | ,, |
| Furniture and equipment 48,000= | 35 16 6 | ,, |
| Total cost ...... £314,500= | £234 14 0 | ,, |

The above calculation is based upon the number of beds in the permanent pavilions, viz., 1,340, although, as I have stated, there is really accommodation at all times for 1,500 patients. It may be remembered that St. Thomas's Hospital cost about £1,000 per bed for land and buildings alone, yet notwithstanding this great outlay, it certainly cannot claim to be so effective for its purposes as the Hamburg Hospital.

A brief summary of important particulars may be of general interest. The medical staff consists of 26 officers, all of whom are fully-qualified men, and they are all paid for their services, a very significant fact. There are seven qualified dispensers, 90 female nurses, and 104 male attendants and servants. The total cost of maintenance for the year 1889 was £53,000, which, allowing for an average of 1,250 patients in residence equals about £43 per bed. When this is compared with the average cost per bed in the London hospitals, one experiences a very uncomfortable surprise. The number of patients treated during 1889 was 11,368.

I have endeavoured to condense this short account of a really remarkably institution, but I would strongly recommend my hearers to avail themselves of the earliest opportunity of paying it a visit of inspection, for I am sure that all who are interested in hospital construction will learn much from such a visit. I found the officials and medical staff most courteous, and ready to show me everything, or to furnish me with all possible information. It was with much regret I learnt that the exceptional advantages afforded by this hospital for teaching purposes are entirely wasted, there being no medical school in Hamburgh, and therefore no students to avail themselves of this wealth of clinical material.

I must confess that I came away feeling somewhat discontented with the general character and equipment of our voluntary hospitals, which in comparison appears so far behind this German State institution in many important respects. I also felt a strong desire to see a really model hospital established on the outskirts of London, for it is admitted by all competent authorities on the subject that a large proportion of patients at present under treatment in our London hospitals, more especially serious surgical cases and those suffering from grave maladies, would do much better if treated in a comparatively pure atmosphere.

I know that there are many and great difficulties in the way of carrying out such a project, but I do not believe that they are insurmountable. The only hope seems to be that one of the great endowed hospitals may establish such a model institution, either on the northern heights or the Surrey hills. Such an outlay would certainly be productive of more benefit to suffering humanity than if expended on further enlargement of hospitals already in many cases far too large to be situated in the midst of the dense population of this great city.

